# Comparison of *De Novo* Transcriptome Assemblers and *k-mer* Strategies Using the Killifish, *Fundulus heteroclitus*

**DOI:** 10.1371/journal.pone.0153104

**Published:** 2016-04-07

**Authors:** Satshil B. Rana, Frank J. Zadlock, Ziping Zhang, Wyatt R. Murphy, Carolyn S. Bentivegna

**Affiliations:** 1 Department of Biological Science, Seton Hall University, South Orange, New Jersey, United States of America; 2 Department of Natural Sciences and Mathematics, SUNY Cobleskill, Cobleskill, New York, United States of America; 3 Department of Chemistry and Biochemistry, Seton Hall University, South Orange, New Jersey, United States of America; University of Western Australia, AUSTRALIA

## Abstract

**Background:**

*De novo* assembly of non-model organism’s transcriptomes has recently been on the rise in concert with the number of *de novo* transcriptome assembly software programs. There is a knowledge gap as to what assembler software or *k-mer* strategy is best for construction of an optimal *de novo* assembly. Additionally, there is a lack of consensus on which evaluation metrics should be used to assess the quality of *de novo* transcriptome assemblies.

**Result:**

Six different assembly strategies were evaluated from four different assemblers. The Trinity assembly was used in its default 25 single *k-mer* value while Bridger, Oases, and SOAPdenovo-Trans were performed with multiple *k-mer* strategies. Bridger, Oases, and SOAPdenovo-Trans used a small multiple k-mer (SMK) strategy consisting of the k-mer lengths of 21, 25, 27, 29, 31, and 33. Additionally, Oases and SOAPdenovo-Trans were performed using a large multiple k-mer (LMK) strategy consisting of k-mer lengths of 25, 35, 45, 55, 65, 75, and 85. Eleven metrics were used to evaluate each assembly strategy including three genome related evaluation metrics (contig number, N50 length, Contigs >1 kb, reads) and eight transcriptome evaluation metrics (mapped back to transcripts (RMBT), number of full length transcripts, number of open reading frames, Detonate RSEM-EVAL score, and percent alignment to the southern platyfish, Amazon molly, BUSCO and CEGMA databases). The assembly strategy that performed the best, that is it was within the top three of each evaluation metric, was the Bridger assembly (10 of 11) followed by the Oases SMK assembly (8 of 11), the Oases LMK assembly (6 of 11), the Trinity assembly (4 of 11), the SOAP LMK assembly (4 of 11), and the SOAP SMK assembly (3 of 11).

**Conclusion:**

This study provides an in-depth multi *k-mer* strategy investigation concluding that the assembler itself had a greater impact than *k-mer* size regardless of the strategy employed. Additionally, the comprehensive performance transcriptome evaluation metrics utilized in this study identified the need for choosing metrics centered on user defined research goals. Based on the evaluation metrics performed, the Bridger assembly was able to construct the best assembly of the testis transcriptome in *Fundulus heteroclitus*.

## Introduction

Next generation sequencing (NGS) technologies have offered unprecedented opportunities to obtain genetic information for non-model organisms with little or no molecular information available [1]. This increasingly accessible technology provides an efficient and cost-effective approach for analyzing the transcriptome of non-model organisms that lack a fully-sequenced genome [2–6]. It has been employed to identify novel transcriptome sequences, single nucleotide polymorphisms (SNPs), simple sequence repeats (SSRs), splicing variants, transcript isoforms, new large intergenic noncoding RNAs, and relative levels of transcript expressions [1–2, 6–7]. With the arrival of NGS technologies, the number of publications characterizing *de novo* assemblies for non-model organisms have steadily been on the rise [8].

*De novo* transcriptome assembly is performed by taking the enormous amount of short read sequences produced by NGS and overlapping them to form contiguous sequences (contigs) [9]. The quality of the assembly output is reliant on the user designated *k-mer* value defined as the sequence overlap between two reads forming the contig [9–12]. Low *k-mer* values have a tendency to recover less abundant transcripts, while producing a large amount of contigs, with a number of them highly fragmented due to sequencing errors and lack of overlap [11, 13]. High *k-mer* values will produce a more contiguous assembly consisting of high coverage transcripts and splice variants. However, the assembly will contain fewer contigs leading to lower transcript representation [11, 13]. Therefore, utilizing a single *k-mer* approach when performing a *de novo* assembly can result in loss of relevant biological information due to the lack of transcript diversity [13]. A logical approach to resolve this dilemma is to cluster multiple single *k-mer* assembles together in order to take advantage of the characteristics of both the low and high *k-mer* values and thereby improve the accuracy of the assembly [9–11]. Investigations that compare the quality of transcriptome assembly using one or multiple *k-mer* values will contribute to standardizing NGS data processing.

The number of *de novo* transcriptome programs developed for assembly of short sequence reads has increased within the past few years. In 2010, Trans-AByss was reported to have the ability to merge multiple individual *k-mer* assemblies, allowing the transcriptome to be represented by wide levels of transcript expression [12]. In 2011, Trinity was reported to be able to fully reconstruct a large fraction of transcripts with low base error rates and have the ability to report alternative splice isoforms [14]. Trinity is currently regarded to be the best single *k-mer* assembler [15]. In 2012, Oases was reported to improve significantly on the Trans-ABySS and Trinity assemblers by merging the use of multiple k-mers presented in Trans-AByss with a topological analysis similar to that presented by Trinity [16]. In 2014, SOAPdenovo-Trans was reported to be able to perform multiple individual *k-mer* assemblies and provide higher contiguity, lower redundancy and faster execution when compared to Trinity and Oases [17]. All of these assemblers are founded on the *de Bruijn* graph-based assembly method to which programmers add their own algorithms [12, 14, 16–17]. In 2015, Bridger, which employs a new *de novo* assembly method that does not construct *de Bruijn* graphs, was created [18]. This assembler uses a rigorous mathematical model, called the minimum path cover, to construct splice graphs that are used to build compatibility graphs for transcriptome reconstruction from short RNA-seq reads [18]. This multiple *k-mer* assembler aims to build a bridge between the key concepts of two popular assemblers, the reference-based assembler, Cufflinks [19], and the *de novo* assembler, Trinity [18]. Given the number of programs available, there is a need for more definitive information on what assemblers and parameters work best for constructing a *de novo* transcriptome [10, 20].

Much effort has been dedicated towards improving the capabilities of *de novo* assembly software. However, methods to evaluate the assembler’s performance are still a few steps behind. For example, a common approach to assess genomes assemblies is to evaluate statistics such as the number of contigs, the amount of contigs over 1,000 bps, and the N50 value. The N50 value is defined as the length of the largest contig from all the contigs ranked smallest to largest that represents 50% of the assembly length [21–22]. However, these metrics are also routinely used to evaluate the quality of *de novo* transcriptome assemblies even though they may be misleading regarding their accuracy [21–23]. Evaluating mRNA characteristics such as the percentage of assembled full length transcripts and the number of long open reading frames (ORFs) are other common metrics for evaluating transcriptome assemblies [13, 15, 24]. A novel reference-free evaluation method to assess the quality of transcriptomes is Detonate RSEM-EVAL [21]. This program produces a statistically principled evaluation score using multiple factors, such as the compactness of the assembly and its support from the RNA-Seq reads used to create it [21].

Annotation-based metrics describe the percentage of sequences within an assembly that match protein sequences found in a related species or curated database [25]. An accurate assembly should contain a high percentage of these conserved proteins while a low percentage reflects mis-assemblies. These types of metrics can be challenging for non-model species that do not have an annotated phylogenetically related species to which it can be aligned. If the evolutionary distance between the two species is too great, then orthologs may have undergone nucleotide changes making alignments less likely to occur [23]. A database to gauge the performance of an assembly strategy in the absence of a well annotated phylogenetically related species is the CEGMA (Core Eukaryotic Genes Mapping Approach) database [26]. This is a manually curated database that contains 248 core proteins present in a wide range of taxa [26]. The same approach can be achieved by utilizing the manually curated BUSCO (Benchmarking Universal Single-Copy Orthologs) protein set for the quantitative assessment of transcriptome assembly and annotation completeness [27]. Using this database, assemblies are matched to 3,023 vertebrate genes and results return the percentage of unaligned sequences. Annotation metrics are useful for assessing both genome and transcriptome assemblies.

Evaluation metrics are important for assessing the quality of genome and transcriptome assemblies. Unfortunately, there is a lack of consensus as to which evaluation metrics work best let alone how many of them to use. For example, Chropra *et al*. performed a comparison study using peanut (*Arachis* spp.) RNA-Seq data. Assemblies were evaluated based on the N50 length, average contig length, number of contigs, the novelty of each assembly using the Mummer tool, the accuracy determined by RMBT, and the continuity by estimating the number of full length transcripts [13]. Moreton *et al*. relied on the RMBT and CEGMA percentages as well as the N50 length, the number of transcripts, and the number of transcripts >1kb when evaluating different assemblies of the duck (*Amas platyrhynchos*) [10]. He *et al*. assessed the assemblies of sweet potato fungus (*Trametes gallica*) and wild rice (*Oryza meyeriana*) based on N50 length, average contig length, number of contigs > 1,000 bps, as well as their ORFs and percent annotation using BLASTX results against phylogenetically related species [15]. More information on which evaluation metrics best predict the quality of *de novo* transcriptome assemblies would help establish “best practices” particularly for less experienced users.

The study presented compares assembly programs, *k-mer* strategies, and various metrics for determining *de novo* transcriptome assembly quality. Assembly performance is evaluated using the testis transcriptome of *Fundulus heteroclitus*, an estuary fish that is a sentinel teleost species commonly used in environmental toxicology studies [28]. To date, this is the first study to compare the performances of the commonly used *de Bruijn* graph-based *de novo* assemblers, Trinity, Oases, and SOAPdenovo-Trans, with a new *de novo* method employed by Bridger. This is also the first study to evaluate the effects of using a small and large multiple *k-mer* strategy for the Oases and SOAPdenovo-Trans assemblers within the same study. Based on the eleven evaluation metrics presented above, it was found that the product of those assemblies was more influenced by the assembler itself than the *k-mer* strategy. Overall, Bridger performed more often within the top three of each evaluation metric than the *de Bruijn* graph-based programs for the *de novo* transcriptome assembly of the killifish RNA-Seq reads.

## Materials and Methods

### Fish Collection and Nucleic Acid Isolation

Male fish identified as Atlantic killifish (*Fundulus heteroclitus*) were collected from Tuckerton, NJ (Little Sheepshead Creek) using baited minnow traps. The authority who issued the permission for capturing the killifish was The New Jersey Department of Environmental Protection; Division of Fish and Wildlife (Permit #1125). The vertebrate work done in this study was approved by The Rutgers University’s Institutional Animal Care and Use Committee (IACUC) (Protocol #08–025). In all instances the fish were alive when captured, and capture methods followed approved animal handling protocols. The fish were transported immediately back to Rutgers University in aerated containers containing water from the collection site to reduce stress. The fish were housed under laboratory conditions for one week before they were sacrificed with an overdose of MS-222 (tricaine methanesulphonate) followed by spinal cord dislocation. The testis were dissected and stored in RNAlater (Qiagen) at -20°C prior to RNA extraction.

Total RNA was extracted from the testis using TRIzol® Kit (Invitrogen™) following the manufacturer’s instructions. Potential genomic DNA contamination in the RNA sample was removed by DNase I digestion (Ambion, Inc.). Using a NanoDrop spectrophotometer (Thermo Fisher Scientific, Inc.), the RNA was quantified by measuring the absorbance at 260 nm. The purity of the RNA sample was assessed at an absorbance ratio of 260/280. The RNA integrity number (RIN) was measured with a BioAnalyzer 2100 (Agilent), for a RIN value > 7.0.

### Illumina Short-Read Library Construction and Sequencing

Ribosomal depletion and mRNA selection was performed with MicroPolyA purist (Ambion). The mRNA was quantified and ribosomal RNA fractions under 2% were verified using a BioAnalyzer mRNA Nano Kit (Agilent). The dUTP-strand specific cDNA library was made using chemical hydrolysis and the Illumina Ultra Directional RNA-seq kit (NEB). Libraries were barcoded with Tru-Seq adaptors and amplified with 12–15 cycles of PCR (Illumina). Completed RNAseq libraries were quantified using Qubit DNA HS, BioAnalyzer High Sensitivity DNA (Invitrogen, Agilent, KAPA). The libraries were sequenced using the NextSeq 500 High Output 300 cycles kit, reading 155 x 155 bp Paired End Sequencing.

### Sequence Data Processing

The quality of the raw Illumina sequence reads were initially assessed using FastQC v0.10.1 [29]. Based on the analysis report, Trimmomatic v0.32 was used to remove all the low quality reads with a Phred score below 20 as well as the Illumina adapters [30]. Contaminating sequences were removed from the reads by using Deconseq with the parameters set to 90% of the contig length with an identity of 94% [31]. FastQC was performed again to verify the integrity of the remaining raw Illumina sequence reads. Upon completion, the quality assessed reads were then ready to be used as the input for the various assembly strategies.

### *De novo* Transcriptome Assembly

Six different assembly strategies were created from four different assemblers: Trinity (v2.0.6), Velvet (v1.2.07) and Oases (v0.2.08), SOAPdenovo-Trans (v1.03), and Bridger [14, 16–18, 32]. The Trinity assembly was the only single *k-mer* assembly. It was run with its default *k-mer* value of 25. The Bridger, Oases, and SOAPdenovo-Trans assemblies were performed with multiple *k-mer* strategies. Bridger, Oases, and SOAPdenovo-Trans used a small multiple *k-mer* (SMK) strategy consisting of the *k-mer* lengths of 21, 25, 27, 29, 31, and 33. The SMK strategy was based on the limitations of the Bridger assembler, which can only use *k-mers* values up to 33. Additionally, Oases and SOAPdenovo-Trans were performed using a large multiple k-mer (LMK) strategy consisting of *k-mer* lengths of 25, 35, 45, 55, 65, 75, and 85. All six assembly strategies incorporated the *k-mer* value of 25 to better compare their performances amongst each other. For the multiple *k-mer* strategies used in the Oases, Bridger, and SOAPdenovo-Trans assemblies, all seven individual *k-mer* assemblies from each group were concatenated followed by CD-HIT-EST (v 4.6.1) to further remove the redundancy and to cluster the contigs for annotation [33].

### Statistics of Assemblies

The six different assembly strategies were assessed using typical statistics for the evaluation of *de novo* genome assemblies. These included the total number of contigs produced, each assemblies N50 length, and the amount of contigs over 1,000 bps long. These statistics were determined using Transrate (v1.0.0 beta3) http://hibberdlab.com/transrate/.

### RMBT Analysis

The accuracy of each assembly was assessed by determining the percentage of raw reads that could be mapped back to transcripts (RMBT). First, indexes were generated using bowtie2-build [34]. Then Bowtie2 (v 2.2.5) was used to map the reads against each assembly and provide the metric of accuracy, which is the percentage of raw reads that align [34].

### Phylogenetic Tree Alignments

Another common assessment tool to evaluate the quality of *de novo* transcriptome assemblies is to align the assembled contigs to a well annotated phylogenetically related species. Quality is based on the percentage of contigs that match the protein sequences of the related species. To determine which closely related fish genome to use as a reference, PhyloT (http://phylot.biobyte.de/contact.html) was used. This program generated a phylogenetic tree between killifish and the eleven publically available fish genomes on Ensembl: Amazon molly (*Poecilia Formosa*), Mexican tetra (*Astyanax mexicanus*), Atlantic cod (*Gadus morhua*), Japanese pufferfish (*Takifugu rubripes*), medaka (*Oryzias latipes*), southern platyfish (*Xiphophorus maculatus*), spotted gar (*Lepisosteus oculatus*), stickleback (*Gasterosteus aculeatus*), green spotted pufferfish (*Tetraodon nigroviridis*) Nile tilapia (*Oreochromis niloticus*), and zebrafish (*Danio rerio*). This program creates trees based on the NCBI taxonomy database, and it was visualized by the web based tool, Interactive Tree of Life (v2) [35]. Based on the created phylogenetic tree, southern platyfish (*X*. *maculatus*) and Amazon molly (*P*. *formosa*) were shown to be the closest relative to killifish. Therefore, the six different assemblies were aligned to the Ensembl proteins of southern platyfish and Amazon molly using BLASTX with an E-value cut-off of 1e-3 to quantify the percentage of previously annotated genes found in each assembly.

### CEGMA and BUSCO Alignments

CEGMA (v.2.5) and BUSCO (v1.1) are other reference-based tools for assessing the degree of annotation. They were individually employed to quantitate assembly completion based on the percentage of contigs that do or do not (therefore mis-assembled) align to highly conserved proteins [28].

### Full Length Transcript Analysis

The number of full length transcripts was quantified to further evaluate the performance of each assembly by following scripts provided by the Trinity software package (http://trinityrnaseq.sourceforge.net/). A modified ‘BLASTX’ script was used to calculate each assembly’s alignment coverage to the curator-evaluated database, SwissProt. Full length transcripts were defined in this study by having > 70% alignment coverage and > 90% alignment coverage to SwissProt proteins.

### Open Reading Frames Analysis

The presence of long open reading frames (ORFs) was analyzed to determine the quality of each assembly by using scripts provided by TransDecoder (http://transdecoder.github.io/). BlastP (v 2.2.30+) was used to search the protein database SwissProt with an E-value cut-off of 1e-3. ORFs ranging from >799 bps, >999 bps, and >1,199 bps were determined using gawk to filter the Fasta files by length.

### Detonate RSEM-EVAL Score

DETONATE’s RSEM-EVAL was used to evaluate the quality of the six different assemblies. This program offers a reference-free evaluation method that relies only on the assembly and the reads used to create it [22].

## Results

### Sequencing and *De Novo* Transcriptome Assembly

To globally profile the testis transcriptome of killifish, we employed Illumina NextSeq 500 technology to sequence the libraries generating 7,197,900 pair-end short reads encoding 1,033,683,143 bases (Table 1). All the raw sequencing reads were deposited into the Short Read Archive (SRA) of the National Center for Biotechnology Information (NCBI) and can be accessed under the accession number SRX1058750. To perform quality control of the raw sequence reads, they were processed to remove Illumina adaptor sequences, low quality reads with a Phred score value less than 20, and contaminating sequences. The processing of the raw sequence reads resulted in 6,349,606 (88.21%) clean reads that were assembled by the following assembly strategies; SOAP LMK, SOAP SMK, Oases LMK, Oases SMK, Bridger, and Trinity (Table 1).

**Table 1:** Statistics of the raw reads after Illumina sequencing and processing.

	Killifish
Number of nucleotide bases	1,033,683,143
Number of raw reads	7,197,900
Number of clean reads for assembly	6,349,606
Percent of used reads for assembly	88.21%

### Statistics of Assembly

The statistics of each assembly was initially used to evaluate the performance of each assembly strategy.

Assemblies with the highest to lowest amount of contigs produced were: Bridger > SOAP LMK > SOAP SMK > Trinity > Oases SMK > Oases LMK (Table 2). Assemblies with the longest to shortest N50 length were: Oases SMK > Oases LMK > Bridger > Trinity > SOAP SMK > SOAP LMK (Table 2). Assemblies with the highest to lowest average contig length were: Oases SMK > Oases LMK > Bridger > Trinity > SOAP SMK> SOAP LMK (Table 2). Assemblies with the highest to lowest amount of contigs over 1kb were: Bridger > Oases SMK > SOAP SMK >Trinity > Oases LMK > SOAP LMK (Table 2). In summary, the Bridger assembly produced the most amount of contigs and contigs over 1kb. The Oases SMK assembly produced the longest N50 length and had the highest average contig length. The SOAP LMK assembly performed the worst for the N50 length, most amount of contigs over 1kb, and the average contig length. The Oases LMK assembly produced the least amount of contigs.

**Table 2:** Statistics of the Assemblies.

	SOAP LMK	SOAP SMK	Oases LMK	Oases SMK	Bridger	Trinity
Contig Number	198,085	187,104	99,567	135,312	303,906	180,658
N50 Length	917	1,042	1,676	1,743	1,668	1,189
Minimum Contig Length	200	200	200	200	201	224
Largest Contig Length	11,658	11,658	16,019	21,489	15,151	11,023
Average contig length	622	672	1,021	1,035	879	711
Contigs Over 1k	33,326	36,136	33,847	45,992	81,769	35,527
RMBT	83.78%	80.77%	85.28%	84.18%	87.71%	82.96%

### RMBT Analysis

A common method to evaluate the accuracy of a *de novo* assembly without a reference genome is to determine the percentage of reads that can be mapped back to transcripts (RMBT) constructed by the assembler. Based on this metric, the Bridger assembly had the highest RMBT percentage (87.71%) and the SOAP SMK assembly had the lowest (80.77%). Results for Trinity, SOAP LMK, Oases SMK, and Oases LMK were similar with RMBT percentages ranging from 82.96% to 85.28% (Table 2).

### Phylogenetic Tree Alignments

**Fig 1:**
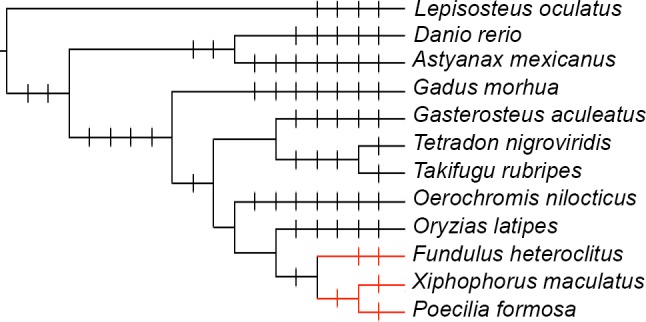
Phylogenetic tree analysis. A phylogenetic tree analysis of the 11 publically available fish genomes and killifish testis. As highlighted in red, the results of Ensembl showed that southern platyfish (*X*. *maculatus*) and Amazon molly (*P*. *Formosa*) are the closest relatives to killifish (*F*. *heteroclitus*).

A strategy to gauge the credibility of a *de novo* assembly is to see how well its annotated sequences compare to those of a related species. Therefore, a phylogenetic tree of killifish and eleven publically available fish genomes was constructed using Ensembl. Based on the results, the southern platyfish and Amazon molly were determined to be the closest related genera to killifish (Fig 1). Therefore the contigs produced from each assembly strategy were aligned to the southern platyfish and Amazon molly genomes independently using BLASTX with an E-value of <1e-3. Results showed that the Oases LMK assembly had the best percentage of alignments to both the southern platyfish (50.14%) and Amazon molly (51.82%) databases (Table 3). The Trinity assembly had the lowest alignment percentage to both the southern platyfish (32.68%) and Amazon molly (34.15%) databases. Results for SOAP LMK, SOAP SMK, Bridger and Trinity were similar with alignment percentages ranging from 32.68 to 37.88% for southern platyfish and 34.15 to 39.35% for Amazon molly (Table 3).

**Table 3:** BLASTX alignments from the six different assemblies against the southern platyfish and Amazon molly databases.

Database	SOAP LMK	SOAP SMK	Oases LMK	Oases SMK	Bridger	Trinity
southern platyfish	34.60%	35.22%	50.14%	47.00%	37.88%	32.68%
Amazon molly	36.46%	37.19%	51.82%	48.71%	39.35%	34.15%

### CEGMA Alignments

A reference-based approach for assessing the quality of an assembly is to align the contigs to the 248 highly conserved proteins in the CEGMA dataset. All of the CEGMA proteins were present in the killifish testis transcriptome. However, none of the assembly strategies were able to incorporate all of them as seen in Table 4. Full length CEGMA proteins, defined as having at least 70% of the protein length found in the CEGMA dataset, ranged from 97.58 to 98.79%. Partial CEGMA proteins ranged from 99.60 to 100%. The Oases LMK and Oases SMK assemblies contained the highest percentage of full length (98.79%) and partial length (100%) CEGMA proteins. The Trinity assembly contained the lowest percentage (97.58%) of full length CEGMA proteins as well as the lowest percentage (98.79%) of partial CEGMA proteins (Table 4). Both of the SMK and LMK strategies of SOAP and Oases were unable to assemble the same CEGMA proteins at full or partial length (Table 4).

**Table 4:** BLASTX alignments of the six different assemblies to the CEGMA dataset.

Assembly	CEGS	CEGs Missing	Partials	Partial Missing
SOAP LMK	98.39%	KOG0261, KOG0209, KOG0462, KOG2311	99.60%	KOG2311
SOAP SMK	98.39%	KOG0261, KOG0209, KOG0462, KOG2311	99.60%	KOG2311
Oases LMK	98.79%	KOG0292, KOG2311, KOG4392	100%	
Oases SMK	98.79%	KOG0292, KOG2311, KOG4392	100%	
Bridger	98.39%	KOG0261, KOG0292, KOG0969, KOG2311	99.60%	KOG0969
Trinity	97.58%	KOG0292, KOG0209, KOG0434, KOG0469, KOG0481, KOG2623	98.79%	KOG0209, KOG0292, KOG0434

### BUSCO Alignments

**Fig 2:**
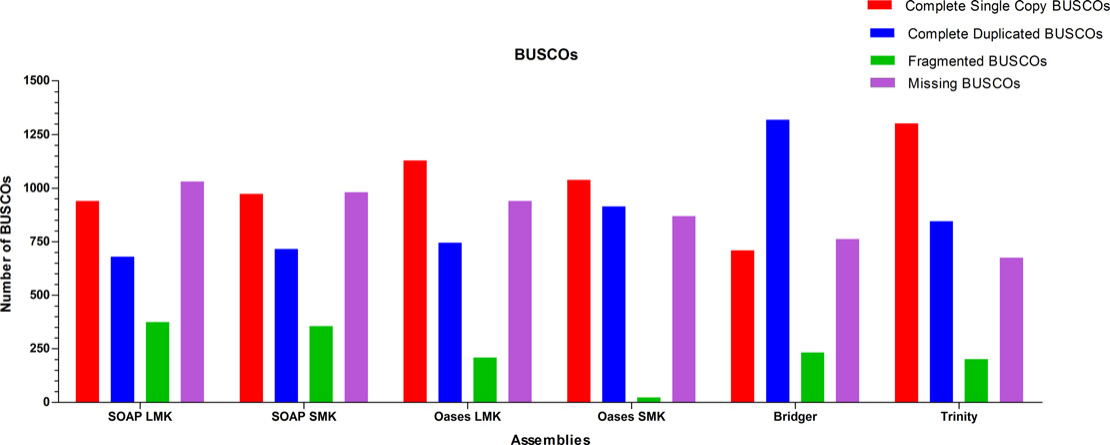
BUSCO Analysis. The Trinity assembly performed the best by having the least amount of missing BUSCOS.

BUSCO is another reference-based program for assessing quality of *de novo* assemblies. The program determined the percentage of mis-assembled transcripts by trying to align all transcripts to highly conserved proteins within the BUSCO dataset. None of the assembly strategies were able to incorporate all of the 3,023 vertebrate BUSCOs genes as seen in Fig 2. Trinity performed best in terms of having the least amount of missing genes 675 (22.3%), followed by Bridger 762 (25.2%), Oases SMK 869 (28.7%), Oases LMK 940 (31%), SOAP SMK 980 (32.4%), and SOAP LMK 1,030 (34.1%).

### Full Length Transcript Analysis

**Fig 3:**
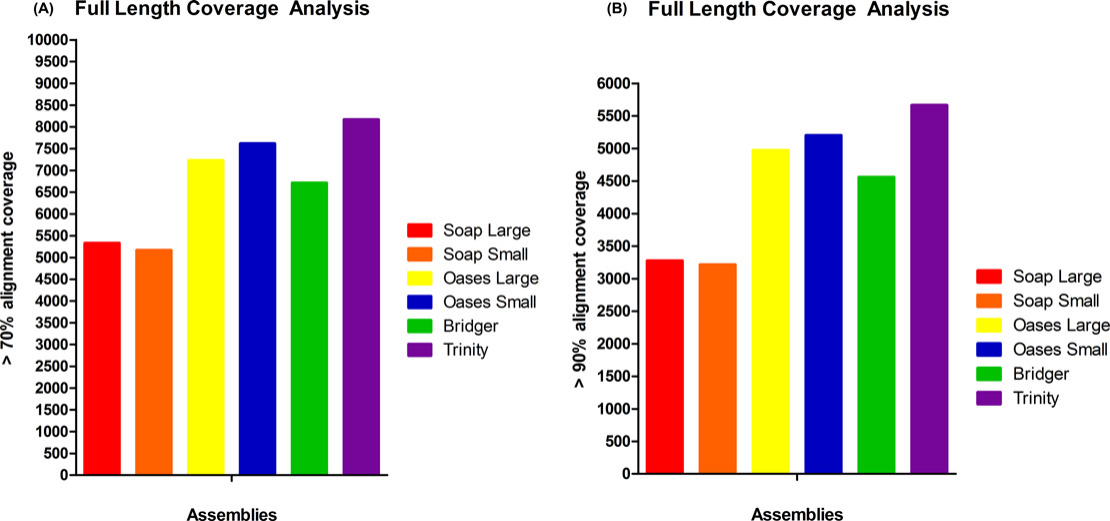
Full Length Transcript Analysis. a) Trinity had the most proteins with greater than 70% alignment coverage. b) Trinity had the most proteins with greater than 90% alignment coverage.

Another metric of assembler performance is quantifying the amount of transcripts that appear to be nearly full length. Based on the alignments with the manually annotated and reviewed SwissProt database, the Trinity assembly performed the best. This assembly produced 8,168 proteins that had greater than 70% alignment coverage and 5,664 proteins that had greater than 90% alignment coverage as seen in Fig 3A and 3B. Other assemblies with the highest to lowest amount of nearly full length proteins were: Oases SMK > Oases LMK > Bridger > SOAP LMK > SOAP SMK. This rank order was the same for 70% and 90% alignment analyses.

### Open Reading Frames Analysis

**Fig 4:**
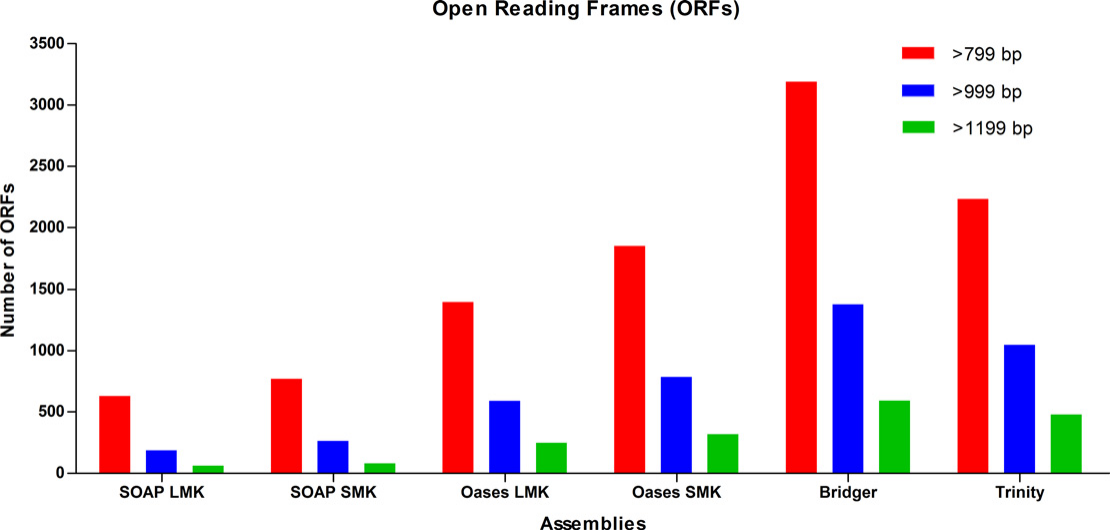
Open Reading Frames Analysis. The Bridger assembly produced the most amount of open reading frames for sequences with >799 bps (red), >999 bps (blue), and >1,199 bps (green).

The contigs in this project were sequenced from mRNA; therefore, the best assembly strategy should produce a large number of open reading frames (ORFs). Overall, the assemblies performed in the same rank order for the presence of ORFs in sequence lengths ranging from >799 bps, >999 bps, and >1,199 bps. Assemblies with the highest to lowest amount of ORFs for each sequence length were: Bridger > Trinity > Oases SMK > Oases LMK > SOAP SMK > SOAP LMK. The Bridger assembly had the highest amount of ORFs for all three lengths. This assembly had 3,189 ORFs > 799 bps, 1,377 ORFs > 999 bps, and 593 ORFs > 1,999 bps (Fig 4). The SOAP LMK assembly had the lowest amount of ORFs for all three lengths. This assembly had 699, 187, and 62 ORFs for sequence lengths ranging from > 799 bps, > 999 bps, and > 1,999 bps, respectively (Fig 4)

### Detonate RSEM-EVAL Score

A novel metric to evaluate the quality of each assembly is Detonate’s RSEM-EVAL score. This score is based on a probabilistic model that only requires the clean reads and the overall assembly for inputs [22]. Assemblies with higher RSEM-EVAL scores are considered better. The assemblies with highest to lowest RSEM-EVAL scores are as follows: Trinity > Bridger > SOAP LMK > Oases LMK > SOAP SMK > Oases SMK (Table 5). The scores for all six assemblies ranged from -5,426.0 x10^6^ to -6,125.0 x10^6^ indicating that all assemblies were very similar (Table 5).

**Table 5:** The Detonate’s RSEM-EVAL scores suggests that the Trinity assembly performed the best. The higher the number value, the better the assembly.

Assembly	SOAP LMK	SOAP SMK	Oases LMK	Oases SMK	Bridger	Trinity
Score	-5,488.0 x10 ˄6	-5,715.0 x10˄6	-5,602.0 x10 ˄6	-6,125.0 x10 ˄6	-5,448.0 x10 ˄6	-5,426.0 x10 ˄6

### Overview of Assembly Strategies

Based on the eleven evaluation metric categories used to assess the six different assembly strategies, it was determined that no one particular assembly strategy performed the best in all categories tested (Table 6). The assembly strategies that performed within the top three of most metrics was the Bridger assembly (10 of 11), followed by the Oases SMK assembly (9 of 11), and then the Oases LMK assembly (6 of 11). The assembly strategies that occurred within the top three the least were the Trinity assembly (5 of 11), followed by the SOAP LMK assembly (4 of 11) and then the SOAP SMK assembly (3 of 11).

**Table 6:** Summary of the top three performers for each evaluation metric category.

	First	Second	Third
Contig Number	Bridger	SOAP LMK	SOAP SMK
N50 Length	Oases SMK	Oases LMK	Bridger
Contigs >1kb	Bridger	Oases SMK	SOAP SMK
RMBT	Bridger	Oases LMK	Oases SMK
southern platyfish DB	Oases LMK	Oases SMK	Bridger
Amazon molly DB	Oases LMK	Oases SMK	Bridger
CEGMA	Oases LMK and Oases SMK	Bridger, SOAP LMK, and SOAP SMK	
BUSCO	Trinity	Bridger	Oases SMK
Full Length Transcripts	Trinity	Oases SMK	Oases LMK
ORFs	Bridger	Trinity	Oases SMK
Detonate	Trinity	Bridger	SOAP LMK

## Discussion

Currently, there is not a gold standard for a *de novo* transcriptome assembler. It is known that different *de novo* assemblers using the same transcripts with similar user defined parameters have produced assemblies that vary amongst each other [10, 13, 15, 25]. One of the goals of this work was to compare four recently published *de novo* assemblers and one user defined parameter, the *k-mer* value, to determine if one strategy was better than another for *de novo* assembly of a non-model organism transcriptome. The assemblers included Trinity, Oases, and SOAPdenovo-Trans (commonly used *de Bruijn* graph-based *de novo* assemblers) as well as Bridger (uses the minimum path cover model to construct splice graphs that are used to build compatibility graphs). Additionally, eleven metrics of assembly quality were compared in order to better form a consensus as to which ones should be used to assess *de novo* transcriptome assemblies. Overall, eleven metrics were used to evaluate six assembly strategies.

Commonly used evaluation statistics, such as the number of contigs, the N50 value, and contig length, were developed for genome assemblies but have also been used to evaluate transcriptome assemblies [22]. Better assemblies should theoretically have more reads assembled into longer contigs and thereby higher N50 values. However, importance of the N50 metric has been questioned [21, 36]. Research indicated that N50 values could be artificially increased based on *k-mer* strategy and or the user defined minimum contig length. Short contigs occurred when high *k-mer* values did not assemble short reads of low abundance transcripts or low *k-mer* values assembled short fragmented transcripts due to lack of overlap [11, 13]. In both cases, if the resulting transcripts were shorter than the minimum contig length parameter established by the user, they were eliminated by the program ultimately generating artificially high N50 values. In the study presented, the influence of high (LMK) and low (SMK) *k-mer* values on evaluation statistics was determined for SOAP and Oases assemblies. Both showed somewhat higher N50 values for low *k-mer* assemblies, but greater differences were found for the assembler. For example, the N50 lengths for SOAP LMK and SMK were 917 and 1,042, respectively, while those for Oases LMK and SMK were 1,676 and 1,743, respectively (Table 2). Bridger also used a low *k-mer* strategy, and results showed that contig number as well as contigs over 1k were twice those of the other assemblies. Taken together, the assembler program had more influence on these traditional, genomic metrics than the *k-mer* values, and the Bridger assembler performed the best in two out of the three metrics.

The quality of an assembly can be assessed by its ability to construct transcripts that align to genes in publically available databases. In the study presented, the six assemblies were evaluated using two well annotated phylogenetic tree relatives, the CEGMA database, and the BUSCO database. For the phylogenetic tree analyses, killifish transcriptome assemblies were aligned to the reference databases of southern platyfish (*X*. *maculatus*) and Amazon molly (*P*. *formosa*) (Fig 1). Oases assemblies had the best percentages of alignments ranging from 47.00 to 51.82%. Results for the other assemblies were similar but lower with alignments ranging from 32.68 to 39.35%. The low percentage of killifish matches in general could be attributed to the evolutionary distance between killifish and the other two species as other *de novo* transcriptome assembly studies using non-model fish had similar findings [37–39]. Additionally, the BLASTX results were based on aligning the testis transcriptome of killifish with the entire genomes of the southern platyfish and Amazon molly. This would have inherently caused a lower percentage of matches. Furthermore, some of the unannotated contigs may be short and consist of sequences lacking well characterized protein domains, comprised of 3’ or 5’ untranslated regions, or be non-coding RNAs [40]. Results also showed that the general performance of each assembly’s BLASTX alignment was more dependent on the assembler software than *k-mer* value. For example, assemblies using Oases SMK and LMK had percent alignments of 47.00 and 50.14% in southern platyfish while SOAP had percent alignments of 35.22% and 34.60% for the same species (Table 3).

CEGMA and BUSCO evaluated assembly quality by aligning contigs to core proteins. For CEGMA, most of the 248 core proteins were found with each assembler; therefore, this metric did not distinguish between them (Table 4). Interestingly, some assemblers left out CEGMA proteins found by others. This indicated that particular assemblers had different proficiencies in reassembling clean reads into transcripts as previously reported by Naksugi et al. [25]. For example, KOG0292 (Vesicle coat complex COPI, alpha subunit) was only present in the SOAP SMK an LMK strategies, and KOG2311 (NAD/FAD-utilizing protein possibly involved in translation) was only present in Trinity. In addition, both SOAP and Oases were unable to properly assemble the same transcripts regardless of the *k-mer* strategy. For example, both SOAP SMK and LMK did not assemble at full length KOG0261, KOG0209, KOG0462 or KOG2311. Both Oases SMK and LMK did not assemble at full length KOG0292, KOG2311 or KOG4392. This indicated that the assembler’s algorithms had a more influential role than the *k-mer* selection when assembling transcripts. BUSCO showed greater differences between assemblers than CEGMA most likely due to the larger database of 3,023 vertebrate genes (Fig 2). Missing BUSCO genes for the six different assemblies ranged from 675 (22.3%) in Trinity to 1,030 (34.1%) in SOAP LMK. As with CEGMA and BLASTX alignments, this metric showed that the assembler rather than the *k-mer* strategy had more influential over the outcome. Both the SMK (28.7%) and LMK (31%) strategies of Oases outperformed the SMK (32.4%) and LMK (34.1%) strategies of SOAP. Overall, results indicated that for either genomes or transcriptomes, if a user’s goal was to annotate the most genes possible for a non-model organism, Oases was the assembler of choice. If the goal was to assemble the most accurate transcriptome, Trinity or Bridger would be the best choice. As of 2015, CEGMA is no longer actively supported and has been essentially replaced by BUSCO.

This study incorporated several evaluation metrics specifically for transcriptomes including number of full length transcripts, ORFs, Detonate’s RSEM-EVAL and RMBT. Shown here as well as other studies, the Trinity assembler proved able to reconstruct the most full length transcripts [13–14, 41]. Alignment coverages were greater than 70% (8,168) and 90% (5,664) even though it utilized a single *k-mer* value (Fig 3A and 3B). The Bridger assembler was able to reconstruct the most ORFs (Fig 4). Results showed that transcripts of >799 bps, >999 bps, and > 1199 bps had 3,189, 1,377 and 593 ORFs, respectively. This number of ORFs was 23.9% (>799 bs), 19.3% (>999 bps), and 16.3% (>1199 bps) higher than those for Trinity, the next closest assembler. SMK and LMK assemblies of Oases and SOAP were similar for both full length transcripts and ORFs; therefore, the overall quality of each assembly's performance was not reliant on the *k-mer* strategy. Detonate’s RSEM-EVAL provided a reference-free evaluation score where a high value indicated an accurate transcriptome assembly. Results showed that the Trinity assembly appeared slightly better than the Bridger and SOAP LMK (Table 5). However, all scores were similar (ranging from 5,426.0 x10 ˄6 to 6,125.0 x10 ˄6); and therefore, this metric did not distinguish well between the assemblers. The RMBT statistic determined assembly accuracy based on the philosophy that the higher the amount of processed reads that can be mapped back to an assembly the fewer the errors (i.e. mis-assembly) introduced by the assembler program. Results for the six assembly strategies evaluated showed RMBT percentages ranging from 87.71% for the Bridger assembly to 80.77% for the SOAP SMK assembly (Table 2). Other studies comparing assembly strategies also reported differences in RMBT percentages [9–10, 13, 20, 25]. In the study presented, the assembler software and not the *k-mer* strategy appeared to have the greater impact. RMBT results showed that the SMK and LMK assemblies for both SOAP (80.77 and 83.78%, respectively) and Oases (84.18 and 85.28%, respectively) performed similarly. Overall, both Trinity and Bridger performed well according to transcriptome-specific metrics and *k-mer* sizes were not a factor.

## Conclusion

This research presents a comprehensive multi *k-mer* strategy assessment with a thorough multiple assembler performance evaluation analysis. To date, this is the first study to compare the performances of the commonly used *de Bruijn* graph-based *de novo* assemblers- Trinity, Oases, and SOAPdenovo-Trans with a new *de novo* method employed by Bridger. Additionally, this is the first study to analyze a small and large multiple *k-mer* strategy for the same assembler software. Based on eleven evaluation metrics, it was found that the assembler had more influence than the *k-mer* strategy. This contradicted Chopra et al., 2014, Chang et al., 2015, and Surget-Groba and Montoya-Burgos, 2010, who highlight the advantages and disadvantages between low and high *k-mer* values (11, 13, 18). For assemblers, killifish data indicated that Bridger performed more often within the top three of each evaluation metric performed better in more of the matrices tested (10 of 11) than the other programs. Oases generated the most alignments with phylogenetically related species. Trinity generated the most full length transcripts, while Bridger generated the most ORFs. Bridger and Trinity also both performed well with BUSCO and Detonate’s RSEM-EVAL. Something that differentiated Trinity and Bridger was time for transcriptome assembly. Bridger was faster, which the authors found to be an important consideration. Other researchers have shown that Trinity takes longer to assemble transcriptomes than other programs including SOAPdenovo-Trans and Oases [17]. Overall, Bridger was the preferred assembler program for producing a quality *de novo* assembly of the killifish testis transcriptome based on its performance within the top three of each evaluation metric along with its time for completion.
